# Investigation of the mechanism of chromium removal in (3-aminopropyl)trimethoxysilane functionalized mesoporous silica

**DOI:** 10.1038/s41598-018-29679-x

**Published:** 2018-08-13

**Authors:** JinHyeong Lee, Jae-Hyun Kim, Keunsu Choi, Hee-Gon Kim, Jeong-Ann Park, So-Hye Cho, Seok Won Hong, Jung-Hyun Lee, Jun Hee Lee, Soonjae Lee, Seung Yong Lee, Jae-Woo Choi

**Affiliations:** 1 Materials Architecturing Research Center, Korea Institute of Science and Technology, Hwarang-ro 14-gil 5, Seongbuk-gu, Seoul, 02792 Republic of Korea; 2 Center for Water Resource Cycle Research, Korea Institute of Science and Technology, Hwarang-ro 14-gil 5, Seongbuk-gu, Seoul, 02792 Republic of Korea; 3 School of Energy and Chemical Engineering, Ulsan National Institute of Science and Technology, UNIST-gil, Ulsan, 44919 Republic of Korea; 4 Department of Chemical and Biological Engineering, Korea University, 145 Anam-ro, Seongbuk-gu, Seoul, 02841 Republic of Korea; 5 Division of Nano & Information Technology, KIST school, Korea University of Science and Technology, Hwarang-ro 14-gil 5, Seongbuk-gu, Seoul, 02792 Republic of Korea; 6 Division of Energy & Environment Technology, KIST school, Korea University of Science and Technology, , Hwarang-ro 14-gil 5, Seongbuk-gu, Seoul, 02792 Republic of Korea; 7 Department of Chemical and Biological Engineering, Korea University, 145, Anam-ro, Seongbuk-gu, Seoul, 02841 Republic of Korea; 8 Department of Earth and Environmental Sciences, Korea University, 145, Anam-ro, Seongbuk-gu, Seoul, 02841 Republic of Korea

## Abstract

We are proposed that a possible mechanism for Cr(VI) removal by functionalized mesoporous silica. Mesoporous silica was functionalized with (3-aminopropyl)trimethoxysilane (APTMS) using the post-synthesis grafting method. The synthesized materials were characterized using transmission electron microscopy (TEM), X-ray diffraction (XRD), N_2_ adsorption-desorption analysis, Fourier-transform infrared (FT-IR), thermogravimetric analyses (TGA), and X-ray photoelectron spectroscopy (XPS) to confirm the pore structure and functionalization of amine groups, and were subsequently used as adsorbents for the removal of Cr(VI) from aqueous solution. As the concentration of APTMS increases from 0.01 M to 0.25 M, the surface area of mesoporous silica decreases from 857.9 m^2^/g to 402.6 m^2^/g. In contrast, Cr(VI) uptake increases from 36.95 mg/g to 83.50 mg/g. This indicates that the enhanced Cr(VI) removal was primarily due to the activity of functional groups. It is thought that the optimum concentration of APTMS for functionalization is approximately 0.05 M. According to XPS data, NH_3_^+^ and protonated NH_2_ from APTMS adsorbed anionic Cr(VI) by electrostatic interaction and changed the solution pH. Equilibrium data are well fitted by Temkin and Sips isotherms. This research shows promising results for the application of amino functionalized mesoporous silica as an adsorbent to removal Cr(VI) from aqueous solution.

## Introduction

Chromium (Cr) is extensively used in various industries, including leather tanning, electroplating, paint processing, and wood preservation. These industries produce large quantities of wastewater containing high concentrations of chromium and discharge this water into the environment^1^. In aqueous environments, chromium generally exists in two stable oxidation states, trivalent chromium (Cr(III)) and hexavalent chromium (Cr(VI)), which have different toxicities, mobilities, and bioavailabilities^2^. Cr(III) is relatively insoluble in aqueous systems and is estimated to be 100 times less toxic than Cr(VI)^3^. In contrast, Cr(VI) is known to be relatively toxic to humans and the environment. Cr(VI) can cause various health problems such as nasal and skin irritation, eardrum perforation, and lung carcinoma^4^.

Several methods have conventionally been applied to treat chromium-contaminated wastewater, including coagulation, chemical precipitation, ion-exchange, membrane separation, electrolysis, and electrodialysis. Of these methods, adsorption is considered one of the most promising processes because of its operational simplicity and economic efficiency^5^. Common adsorbents include activated carbons, iron materials, cellulose, biomass, and polymers^6–12^. Polymeric modified silica composites are an important family of adsorbents that have recently attracted increasing attention because of their advantageous physicochemical properties^13^. Aniline formaldehyde condensate-coated silica gel^14^, poly(ethylene imine)-silica nanospheres^15^, polyacrylamide-silica microspheres^16^, polyaniline/silica gel composite^17^, etc., have been used for the removal of Cr(VI) from aqueous solution. Polymers are an important adsorbent for removing Cr(VI), and their highly branched chains with various functional groups can facilitate selective adsorption of metal ions^18^.

Organic functionalization of silica materials to enhance the Cr(VI) adsorption amount has been reported in several studies (performances for previous studies were summarized at Table S1 in Supplementary). In particular, combining the use of silica with nitrogen-containing organic groups, such as aliphatic amines and aromatic amines, has been widely studied^18^. Mesoporous silicas, such as SBA-1, MCM-41^19^, MCM-48^20^, and SBA-15^21^, have been modified with functional groups. The adsorption behavior of metal ions on a functionalized silica surface depends on the concentration of the functional groups on the surface of the adsorbent, the number of donor groups in the grafted ligand, and the pH of the system^22^. It is known that amine groups can be converted into protonated ions in an acidic solution. Therefore, it is expected that Cr(VI) removal amount will increase as the number of nitrogen atoms in the amine group increases. Di-amine agents, containing two amine groups per molecule, have higher adsorption capacities than mono-amine agents. A previous experiment revealed that only surface amine groups participated in the adsorption of hexavalent chromium. Therefore, an effective method to improve the performance of the Cr(VI) adsorbent is to use mesoporous silica, the surface of which is functionalized by amine groups. 3-aminopropyl trimethoxysilane (APTMS) can easily be grafted to silanol in mesoporous silica. Moreover, since APTMS is lighter than APTES, it has the benefit of improved performance, which should be normalized by adsorbent mass.

The aim of this study was to prepare amino-functionalized mesoporous silicas, and apply them to Cr(VI) removal from aqueous solution in batch systems. Mesoporous silica was synthesized in a simple way, and functionalized using various concentrations of APTMS. The Cr(VI) sorption characteristics of the synthesized silica material were examined under batch conditions. Batch experiments were conducted under various experimental conditions; the loading APTMS amount and the initial Cr(VI) concentration were also varied. Equilibrium isotherms were used to analyze the experimental data. Fourier-transform infrared (FT-IR) spectrometry and X-ray photoelectron spectroscopy (XPS) were used to investigate the Cr(VI) sorption characteristics of the adsorbents.

## Results and Discussion

### Characteristics of MPS

**Figure 1:**
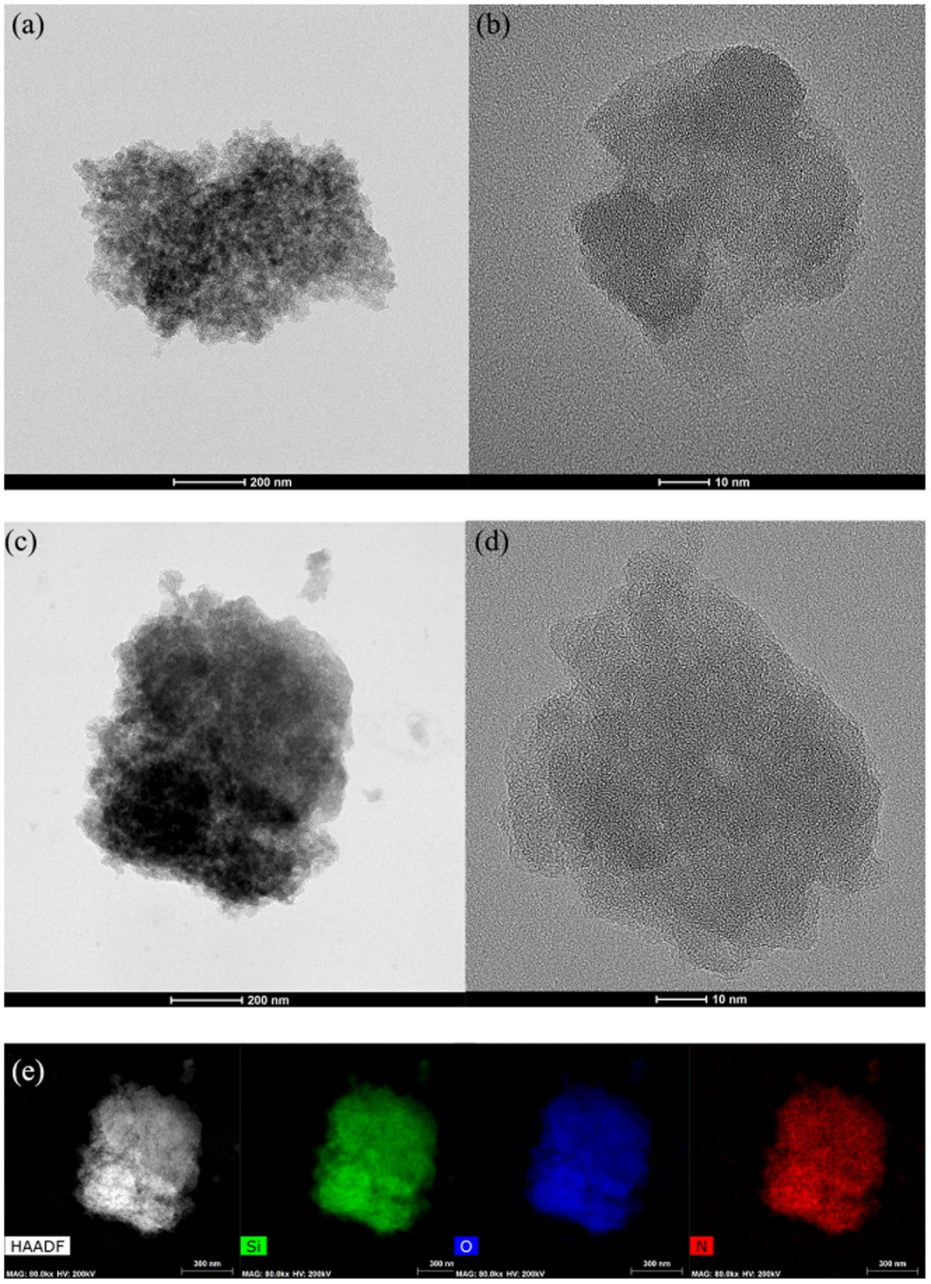
TEM image of mesoporous silica (MPS): (**a**) agglomerates, (**b**) primary particles before APTMS functionalization, (**c**) agglomerates, (**d**) primary particles after APTMS functionalization, and (**e**) TEM–EDS elemental mapping of Si, O, and N for MPS after APTMS functionalization.

TEM observations revealed that the primary MPS particles of 50–60 nm in size formed agglomerates of 700–800 nm in size (Fig. 1). Additionally, the particles did not have a uniform shape. XRD investigations indicated that the particles were not crystalline (Fig. S1 in the Supplementary Material). The compositional analysis in Fig. 1(e) indicates that nitrogen was uniformly distributed throughout the MPS. The results clearly demonstrate that functionalization of APTMS occurred over the entire MPS surface.

**Figure 2:**
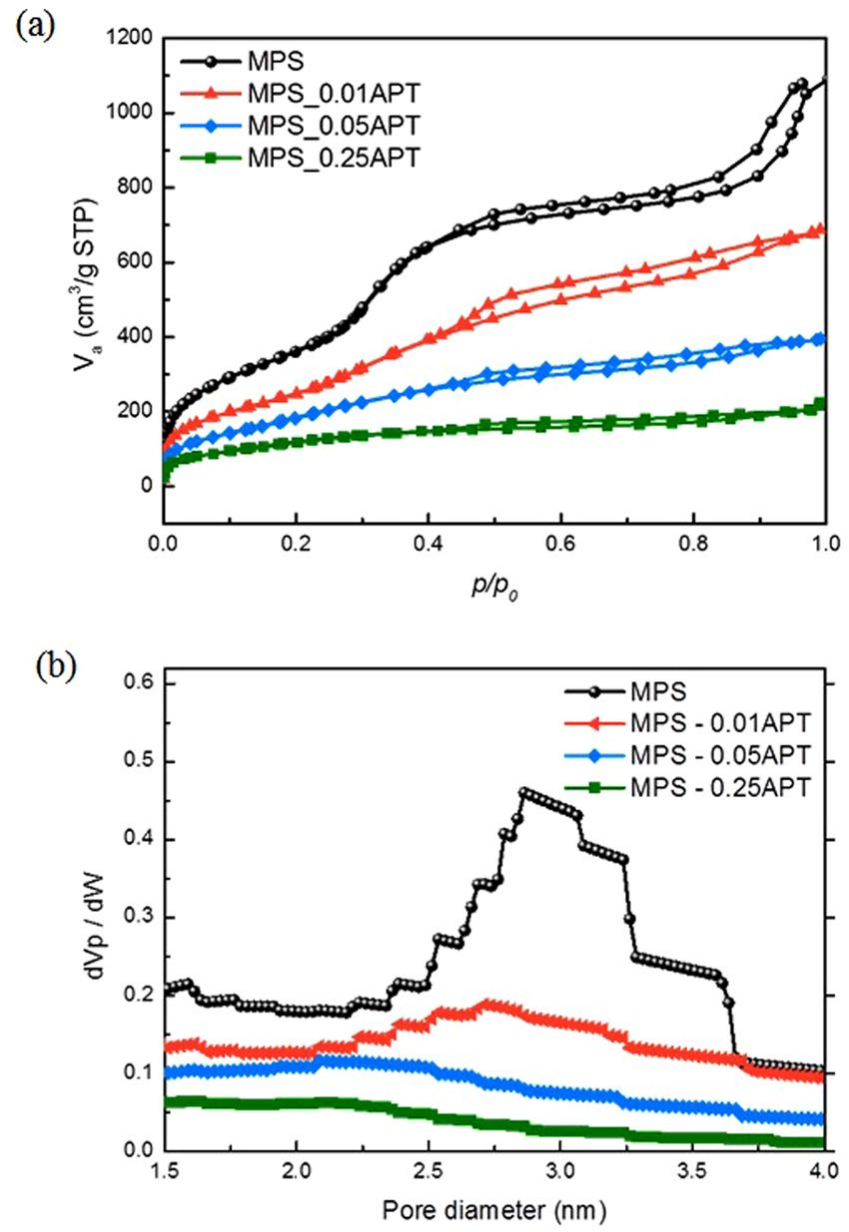
(**a**) N_2_ sorption isotherm graphs for MPS before and after APTMS functionalization and (**b**) pore size distribution of HK plots for MPS.

The MPS exhibits a characteristic type IV BET isotherm graph, shown in Fig. 2(a), indicating a mesoporous structure^23^. It is notable that MPS and functionalized MPS have mesopores as well as micropores, even though they contain more mesopores. (Fig. 2(b)) The Horvath-Kawazoe (HK) plot for MPS has a broad peak at the 2.86 nm pore size. As the concentration of APTMS increases from 0.01 M to 0.25 M, the peak in the HK plot shifts toward smaller pore sizes. This indicates that pores narrowed because a greater number of APTMS molecules interacted on the surface, forming pore walls. This is corroborated by the decrease in the mesopore volume in the Barrett-Joyner-Halenda (BJH) analysis (1.7 cm^3^g^−1^ to 0.256 cm^3^g^−1^) as well as the decrease in the BET surface area (1379.6 m^2^ g^−1^ to 402.6 m^2^ g^−1^) (Table 1).

**Table 1:** Physical characteristics of MPS before/after APTMS functionalization.

	Surface area (m^2^/g)	Mesopore volume (cm^3^/g)	Pore size distribution (nm)
MPS	1379.6	1.7	2.8625
MPS_0.01APT	857.88	1.0698	2.7125
MPS_0.05APT	682.06	0.5492	2.2625
MPS_0.25APT	402.6	0.256	2.0875

**Figure 3:**
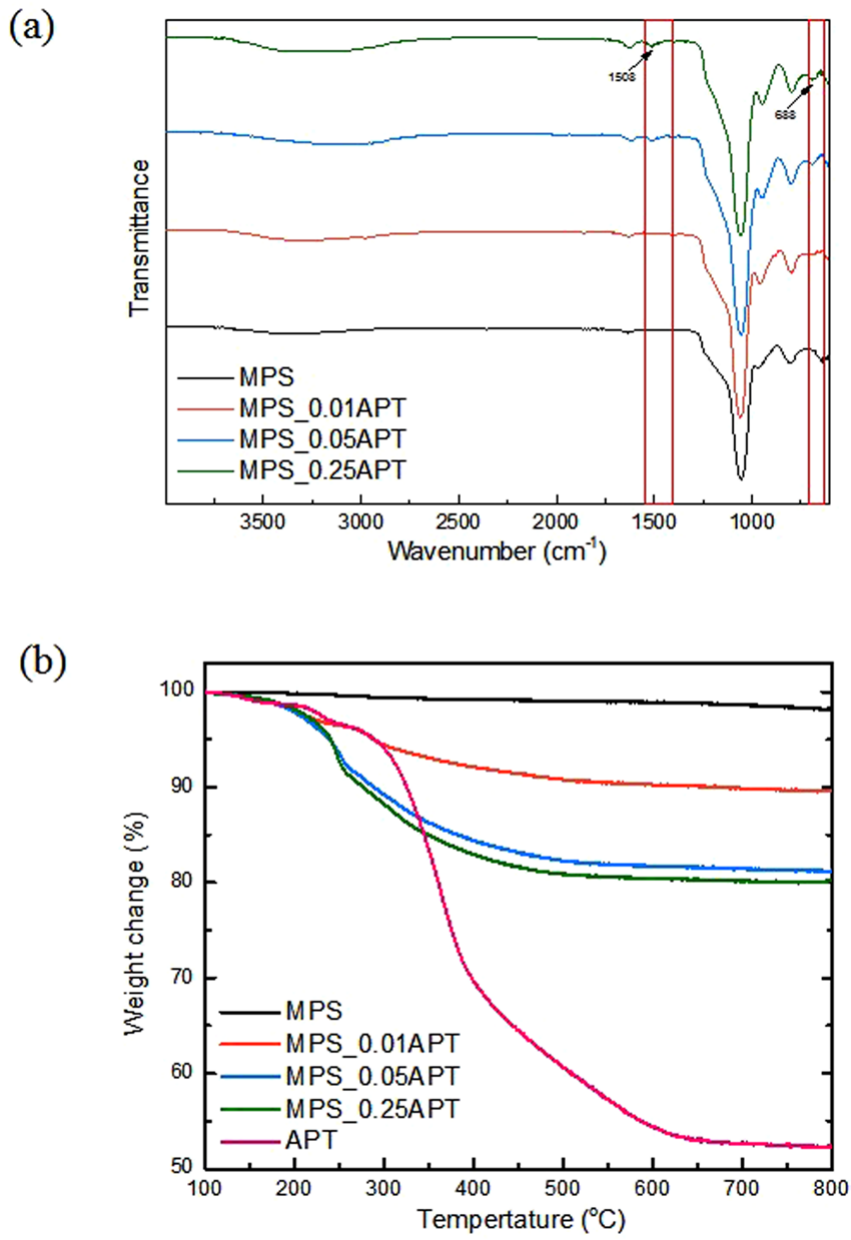
(**a**) FT-IR spectra for MPS before and after APTMS functionalization. (**b**) TGA curves for MPS before and after functionalization with APTMS.

The presence of amino groups in the functionalized MPS structure can be confirmed by FT-IR measurement (Fig. 3(a)). The two weak absorption bands observed around 688 and 1508 cm^−1^ were assigned to the N–H bending vibration and the NH_2_ deformation modes in the primary amides, respectively^24,25^. The spectra clearly show an increase in the peak intensity of the NH_2_ amine group as the concentration of APTMS increases from 0.01 M to 0.05 M. However, the difference in peak intensity between APTMS-2 and APTMS-3 is negligible.

The TG analysis can explain the similarity between APTMS-1, APTMS-2 and APTMS-3 in their FT-IR spectra. Figure 3(b) shows the TGA curves for MPS, functionalized MPS, and APTMS, and shows that the MPS supports have thermal stability with weight loss of <2%. For functionalized MPS, TG curves show increased weight loss with temperature, and the weight loss is proportional to the APTMS loading^26^. The loading amount after APTMS functionalization can be calculated by considering the residual amount of pure APTMS (Table 2). Table 2 shows the difference in the degree of surface functionalization by the concentration of APTMS. As the concentration increases from 0.01 M to 0.05 M, the loading amount increases from 22.2 wt% to 57.1 wt%. However, the loading amount of 63.1 wt% for APTMS-3 is slightly higher than the value for APTMS-2. A further five-fold APTMS concentration increase in the reaction solution did not make a significant difference to the loading amount, probably because the amount of reacted APTMS is saturated at 0.05 M.

**Table 2:** APTMS loading of MPS before and after functionalization.

Sample	Residuals (wt%)	Calculated loadings (wt%)
MPS	98.18	—
MPS_0.01ATP	89.64	18.5
MPS_0.05ATP	81.28	36.6
MPS_0.25ATP	80.21	38.9
APTMS	52.3	—

### Removal of Cr(VI) by functionalized MPS

**Figure 4:**
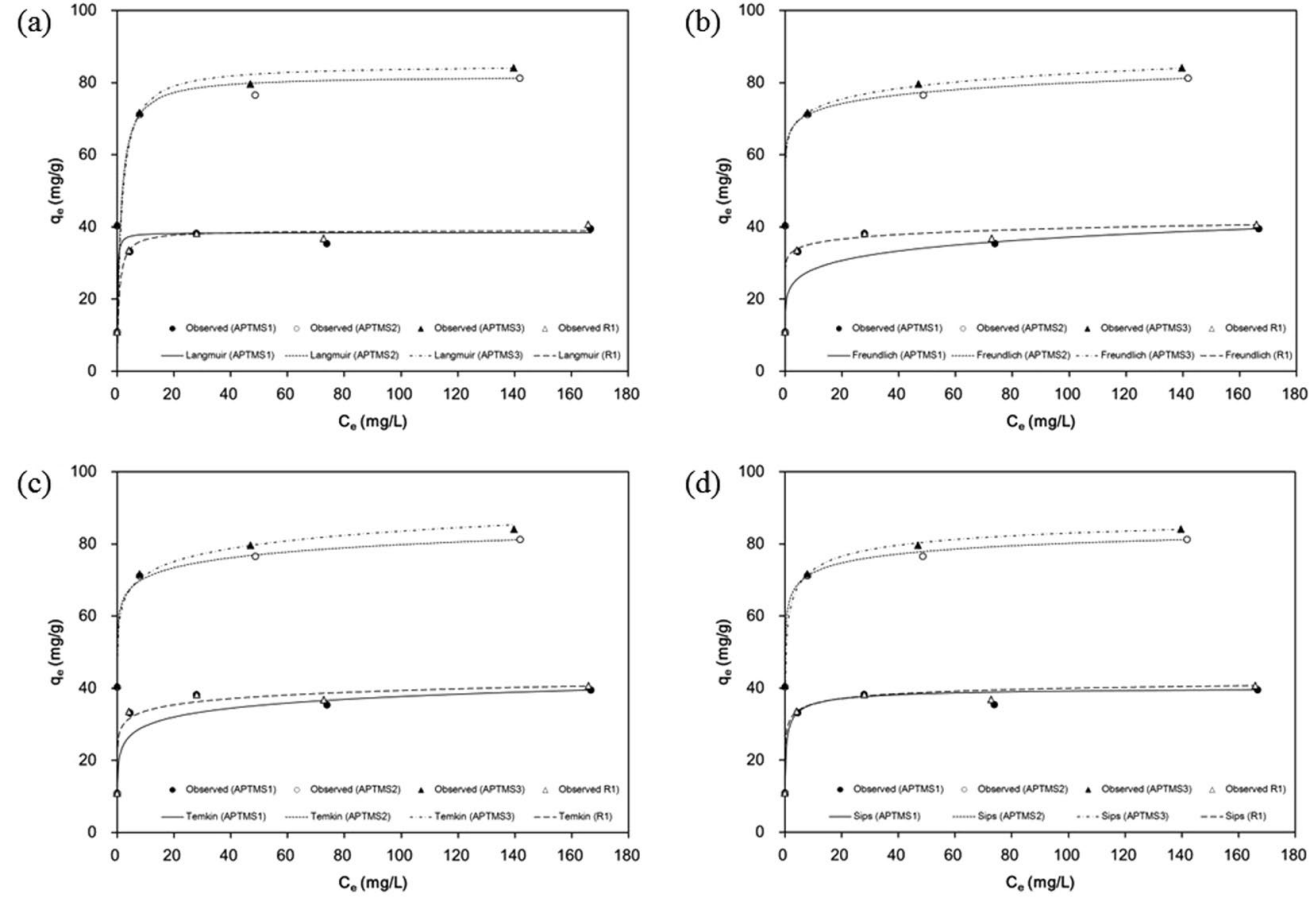
Cr(VI) sorption isotherms for functionalized MPS: (**a**) Langmuir model, (**b**) Freundlich model, (**c**) Temkin model, and (**d**) Sips model.

**Figure 5:**
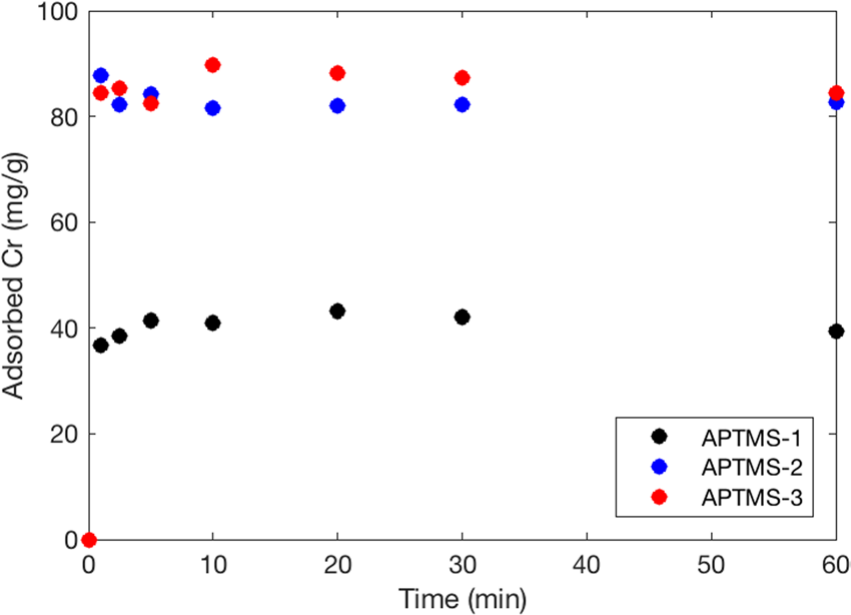
Kinetic test for chromium removal using APTMS-1, -2, and -3 (**a**) chromium concentration in the solution and (**b**) chromium adsorbed on sorbents.

The equilibrium data were fitted using Langmuir, Freundlich, Temkin, and Sips isotherm models (Fig. 4). The corresponding model parameters are provided in Table 3. The values of R^2^ and the sum of absolute error (SAE) indicate that the Temkin isotherm provides the best fit to the equilibrium data, except in the case of APTMS-1, indicating that Cr(VI) adsorption onto functionalized MPS could be a chemisorption process^27^. The positive values of RT/b_T_, the constant related to heat adsorption, indicate that the adsorption process is endothermic in nature^28^. The isotherm data for APTMS-1 is well fit by the Sips model, indicating the high affinity of surface groups for Cr(VI)^29^. Maximum Cr(VI) uptake values (Q_m_) of 39.06, 38.43, 81.89, 84.90 mg/g were obtained from the Langmuir model for MPS, APTMS-1, APTMS-2, and APTMS-3, respectively. These maximum Cr uptake values were consistent with those obtained from the Sips model. Kinetic adsorption test shows that Cr removal using APTMS-1, APTMS-2 and APTMS-3 were occurred instantaneously and finished within 1 min (Fig. 5). This instantaneous reaction was due to the mesoporous structure and the high surface area. Cr removals of APTMS-1, APTMS-2, and APTMS-3 in the kinetic tests were 39.39, 82.57 and 84.34 mg/g at 60 min, consistent with the maximum Cr uptakes in the equilibrium test.

**Table 3:** Equilibrium isotherm model parameters obtained from model fitting of the experimental data.

	Langmuir model				Freundlich model				Temkin model					Sips model				
	Q_m_ (mg/g)	K_L_ (L/mg)	R^2^	SAE	K_F_ (L/g)	1/n	R^2^	SAE	A_T_ (L/g)	b_T_ (J/mol)	RT/b_T_	R^2^	SAE	q_m_ (mg/g)	a_S_	_βS_	R^2^	SAE
APTMS1	38.43	7.81	0.966	8.25	21.49	0.12	0.834	18.46	4.30 × 10^2^	712.90	3.48	0.914	12.75	40.73	1.92	0.56	0.982	4.10
APTMS2	81.89	0.83	0.876	54.71	64.66	0.05	0.877	52.10	5.34 × 10^6^	634.56	3.91	0.988	1.82	97.96	1.72	0.21	0.877	52.66
APTMS3	84.90	0.67	0.883	54.33	63.98	0.06	0.883	52.32	8.22 × 10^4^	480.21	5.16	0.999	2.58	92.80	1.58	0.36	0.883	52.34
R1	39.06	1.42	0.985	14.80	31.65	0.05	0.988	14.64	8.32 × 10^4^	1019.39	2.43	0.800	5.76	45.82	1.80	0.29	0.989	14.07

**Figure 6:**
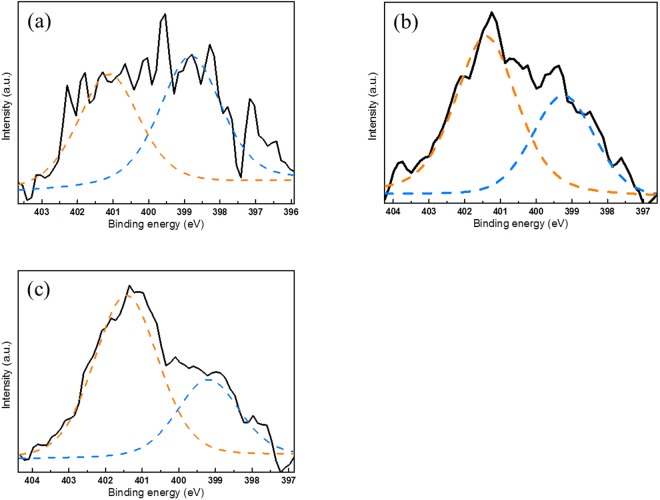
XPS spectra for APTMS-treated silica nanoparticles: (**a**) APTMS-1, (**b**) APTMS-2, and (**c**) APTMS-3 (yellow: -NH_3_^+^, blue: -NH_2_).

The pH of the prepared solutions depends on the Cr(VI) concentration: higher concentrations give lower pH values because the formation of Cr(VI), [HCrO_4_]^−^, releases seven H^+^ ions into solution. Each sample was analyzed using the XPS technique to investigate the chemical bonding and construction of the modified surface. High-resolution XPS spectra were also analyzed as shown in Fig. 6. The higher the APTMS concentration during functionalization, the stronger the peak intensity binding energy near 103 eV. The high-resolution N 1s spectrum consists of two different peaks^30,31^. The binding energy around 398.8 eV corresponds to -NH_2_, and that at 401.3 eV corresponds to -NH_3_^+^. The -NH_3_^+^/-NH_2_ ratio increases as APTMS concentration increases. If functionalization of the MPS surface is not complete at low APTMS concentration, hydroxyl groups are exposed on the surface. These hydroxyl groups can form hydrogen bonds with NH_2_, which makes the subsequent HCl treatment ineffective and induces the low -NH_3_^+^/-NH_2_ ratio for APTMS-1. At high APTMS concentration, pre-existing NH_3_^+^ groups can repel H^+^ ions, and hence the ratio of NH_3_^+^ does not monotonically increase from APTMS-1 to APTMS-3, but is almost saturated at APTMS-2.

Table 1 shows the pore structure information for each sample, and indicates that the BET surface areas are inversely proportional to the APTMS contents. The maximum BET surface area is 857.9 m^2^/g for APTMS-1, and the minimum value is 402.6 m^2^/g for APTMS-3. In contrast, the Cr(VI) uptake increases from 36.95 mg/g for APTMS-1 to 80.12 mg/g for APTMS-2, and shows a slight increase to 83.50 mg/g for APTMS-3, even though the content of APTMS-3 was five times higher than for APTMS-2. These two results indicate that the crucial factor for Cr(VI) adsorption is not the inner pore structure but the introduction of specific functional groups. Considering the composition of silica-APTMS, amine groups, -NH_2_ and -NH_3_^+^, can be adsorption site for Cr(VI), since saturated C atom is not reactive to Cr(VI). Especially, the content of -NH_3_^+^ is proportional to the adsorption amount of Cr(VI) by considering the XPS and Cr removal experiments. Several studies have reported that anionic Cr(VI) is adsorbed by NH_3_^+^ groups via electrostatic interaction^14,32,33^.

**Figure 7:**
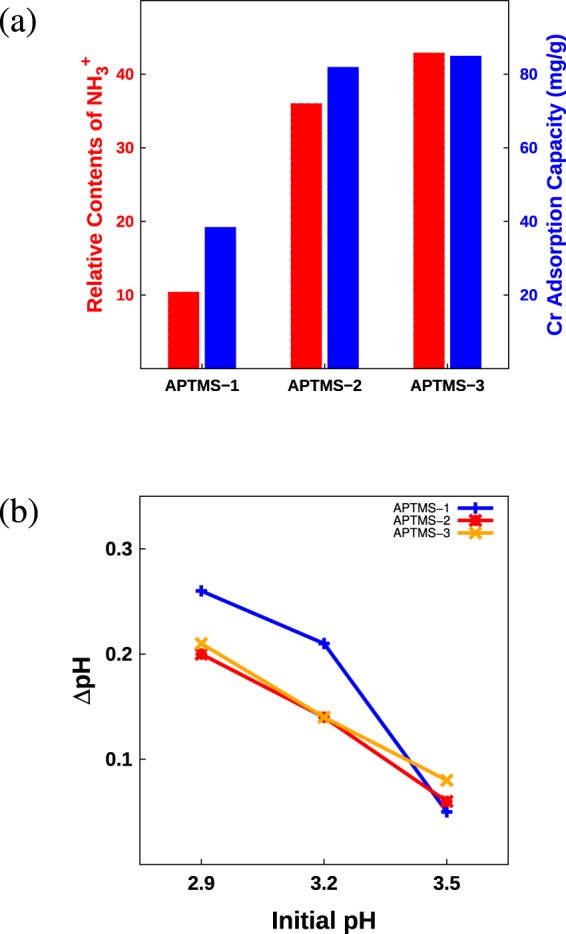
(**a**) Relative contents of NH_3_^+^ and chromium adsorption capacities for APTMS-1, -2, and -3. (**b**) The change in the pH for APTMS-1, -2, and -3 after chromium adsorption for initial pH values of 2.9, 3.2, and 3.5.

To identify the relationship between the contents of NH_3_^+^ and the Cr(VI) adsorption amount, we calculated the relative contents of NH_3_^+^ in APTMS-1, -2, and -3. The loading amounts of APTMS in each sample were 22.2%, 57.1%, and 63.1%, as mentioned above. The -NH_3_^+^/-NH_2_ ratio in APTMS-1, -2, and -3 were 47%, 63%, and 68% according to the XPS N 1 s peak in Fig. 5. Combining this information shows that the relative NH_3_^+^ ratio for the three samples are 10.4:36.0:42.9. However, the Cr(VI) adsorption amount of APTMS-2 was only approximately twice that of APTMS-1, even though APTMS-2 contains -NH_3_^+^ over three times than APTMS-1 as shown in Fig. 7(a). In order to reveal the discrepancy between the adsorption amount of Cr(VI) and the relative content of NH_3_^+^, we investigated the pH changes after chromium adsorption, which are summarized in Fig. 7(b): The higher the initial pH value the smaller the pH change after Cr(VI) adsorption. The relationship between initial pH and pH change is due to the protonation, which combines NH_2_ and H^+^ in the electrolyte to form NH_3_^+^. The protonation is active at low pH by the abundant of H^+^ and is suppressed at high pH by the lack of H^+^. In particular, the pH increase after Cr adsorption at low pH is significantly higher in APTMS-1 than in the other two samples. It might be originated from the high levels of protonation activity at low pH conditions, and the relatively high amounts of NH_2_ in APTMS-1. The additional protonation in APTMS-1 increases the contents of NH_3_^+^, and thus Cr adsorption amount. Relatively small difference in adsorption amount of Cr(VI) compared to the contents of NH_3_^+^ between APTMS-1 and APTMS-2 can be explained by the protonation of NH_2_ depending on the pH condition.

**Figure 8:**
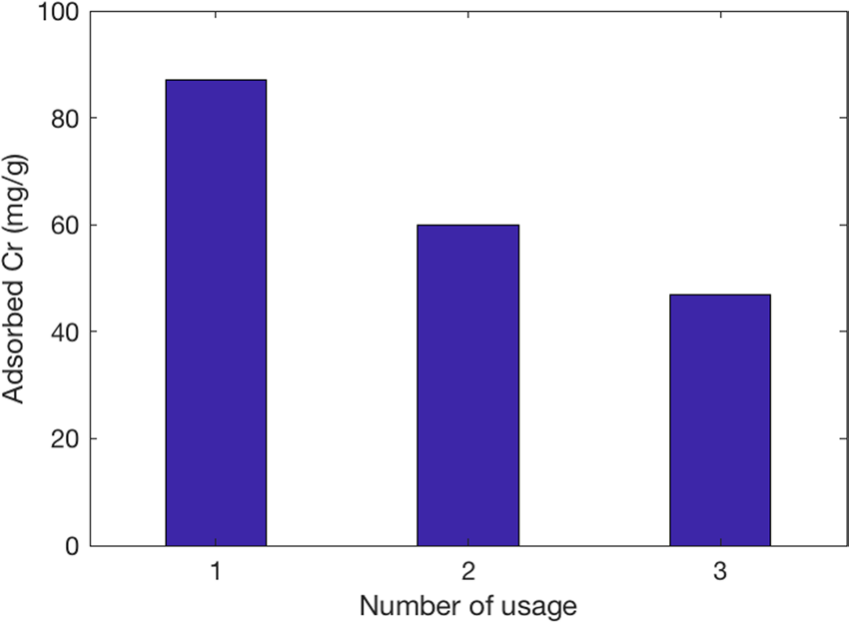
Chromium removal capacity of APTMS -3 regenerated by 0.1 M HCl.

The reusability of an adsorbent with multiple adsorption-desorption processes are important to enhance the cost effectiveness of the adsorption process. It was observed that the chromium adsorption capacity of the APTMS-3 could be regenerated by washing 0.1 M HCl (Fig. 8). This indicates that an acidic condition can facilitate desorption of the chromium from the exhausted adsorbents. The chromium removal capacity was gradually decreased by the repeated regeneration.

## Conclusions

In this study, functionalized mesoporous silicas with amine groups were successfully synthesized using the post-synthesis grafting method. The surface areas of the mesoporous silicas decrease as the APTMS loading increases. According to TG analysis results, the amount of reacted APTMS started to saturate at 0.05 M. The Cr(VI) removal capacities of the synthesized silica materials, MPS, APTMS-1, APTMS-3, and APTMS-5, were investigated. Although the surface areas of the amino-functionalized materials were lower than for the unmodified mesoporous silica (MPS), they showed higher sorption capacities for Cr(VI) compared with MPS. In addition, the Cr(VI) uptake increases from 36.95 mg/g for APTMS-1 to 80.12 mg/g for APTMS-2, but only slightly increases to 83.50 mg/g for APTMS-3. These results indicate that the crucial factor for Cr(VI) adsorption is the introduction of specific functional groups rather than the inner pore structure. However, excessively high densities of amine groups on the mesoporous silica surface can reduce the efficiency of functionalization. Optimum loading amounts of APTMS can induce NH_2_ to protonate completely to NH_3_^+^ through HCl treatment, which plays an important role in Cr(VI) adsorption. Electrostatic interaction between anionic Cr(VI) and NH_3_^+^ groups is a possible mechanism for Cr(VI) sorption by amino-functionalized mesoporous silica. The pH changes in acidic and alkaline environments exhibited different trends for the protonation reaction. Equilibrium data were found to be well represented by Temkin and Sips isotherms. The Cr(VI) uptake does not depend on the pore structure, but on the protonated primary amine group. NH_3_^+^ decreases as the pH increases, and therefore the Cr(VI) uptake also decreases.

## Materials and Methods

### Materials

All chemicals were obtained from commercial suppliers and used without further purification. Tetraethyl orthosilicate (TEOS, 99.999% metal basis), hexadecyltrimethylammonium bromide (CTAB H5882, >98%), branched polyethylene imine (PEI, MW. 800), (3-aminopropyl)trimethoxysilane (APTMS, 97%), ethanol (94.5%), and methanol (≥99.9%) were purchased from Sigma-Aldrich. Hydrochloric acid (HCl) was obtained from DAEJUNG (Korea). In addition, chromium standard solution was also obtained from Kanto Chemicals (Tokyo, Japan). Deionized (DI) water and ethanol were used in all experiments.

### Synthesis of MPS

PEI-silica nanocomposites were synthesized in a simple way. 0.0018 mol of CTAB and 0.0112 mol of PEI were dissolved in 180 mL DI water and 20 mL ethanol, respectively. The PEI solution was mixed with CTAB solution by stirring for 30 min at 800 rpm. Afterwards, 0.0188 mol of TEOS were added as a silica source and the mixture was stirred for 12 h. The product was washed five times in a centrifuge with DI water at 13,500 rpm for 12 min. The washed PEI-silica powder was dried at 70 °C overnight and calcined at 600 °C in air for 12 h to remove the PEI polymer.

### Functionalization of MPS

Functionalization of MPS by APTMS was carried out following a previously reported method with slight modifications^34,35^. The MPS surface was functionalized using various concentrations of APTMS from 0.01 M to 0.25 M. One hundred milliliters of APTMS methanol solution were refluxed via stirring at 1000 rpm at 60 °C for 3 hrs. 1.2 g of MPS were then added to the APTMS solution by stirring at 1000 rpm for 24 hrs. The product was washed with ethanol three times, and dried in a vacuum at 70 °C overnight. After cooling, the product was treated with 0.1 M of HCl for 6 hrs. After 3 times cleaning with centrifugation and drying process, the APTMS-MPS sorbent was prepared.

### Transmission electron microscopy (TEM), X-ray diffraction (XRD), and Brunauer-Emmett-Teller (BET)

Particle sizes and morphologies were observed using TEM (F200X, FEI Company), and compositions were analyzed simultaneously using an F200X-equipped Super-X energy dispersive X-ray spectroscopy (EDS) detector. N_2_ adsorption-desorption analyses were performed using a surface area analyzer (BEL-SORP-max, BEL Japan Inc., Japan). Prior to analysis, the materials were degassed at 100 °C for 2 h to remove moisture. The specific surface area was determined from the linear part of a BET plot (P/P_0_ = 0 to 1). The total pore volume was evaluated from the adsorbed amount at a relative pressure of approximately 0.99.

### Fourier transform infrared (FT-IR) and thermogravimetry analysis (TGA)

FT-IR spectra were obtained by employing a Nicolet iS 10 FT-IR Spectrometer (Thermo Scientific) with a frequency range of 4000 to 600 cm^−1^. Thermogravimetry (TG) analyses were performed using TG apparatus (Scinco N-1500). The samples were heated at 100 °C for 1 hour to remove any moisture, and heated from 100 °C to 800 °C at a rate of 10 °C min^−1^ under air atmosphere.

### X-ray photoelectron spectroscopy (XPS)

XPS was performed on a PHI 5000 VersaProbe Ulvac-PHI (Physical Electronics, Inc.) with an Al X-ray monochromatic source (K_α_ 1486.6 eV at 24.5 W). The binding energy was calibrated to the C 1 s line of adventitious carbon at 284.6 eV.

### Batch experiments for Cr(VI) removal

Chromium removal experiments were conducted under equilibrium batch conditions. The predetermined concentrations of Cr(VI) were prepared by diluting a standard chromium solution. All experiments were performed in duplicate under ambient conditions at a temperature of 25 °C. Each sorption test was conducted using 50 mL conical tubes. First, 50 mL of each chromium solution (the original pH of the metal solutions was 2.91–4.19), with initial concentrations of 10, 25, 50, 100, and 200 mg/L, were added to 50 mL conical tubes containing 0.03 g of APTMS-MPS. Then, the samples were mixed using a rotary shaker at 100 rpm. The liquid phase was subsequently separated from the solution using a 0.45 μm PTFE syringe filter (Millipore, USA). The chromium concentration was measured by inductively coupled plasma-optical emission spectrometry (ICP-OES) on a Perkin-Elmer (Optima 2000 DV) spectrometer at a pump rate of 1.5 mL/min. The solution pH was measured using a pH probe (8302BNUMD, Orion, USA).

The adsorption tests for APTMS-MPS were also conducted under kinetic batch condition using chromium solutions with initial concentration of 100 mg/L. The adsorbent 0.1 g were added in 560 mL Erlenmeyer flasks containing chromium solution and mixed using a rotary shaker. The solution sample was extracted using syringe at 1, 2.5, 5, 10, 20, 30 and 60 min. The chromium concentration in the liquid phase were measured by ICP-OES.

### Regeneration experiments

To demonstrate the reusability of APTMS-MPS adsorbed Cr, desorption and reuse tests were conducted. The adsorption test was performed by reacting 0.05 g of APTMS 3 (with highest adsorption capacity) with 200 mg/L of Cr solution 200 mL. After 6 h of reaction, the adsorbent was recovered from the solution by centrifugation. The recovered adsorbents were regenerated by suspending in the 0.1 M HCl solution. After washing with 0.1 M HCl solution three times, the particle was harvested by centrifugation and used for the following the reuse test. The reuse test was performed using the same method.

### Adsorption data analysis

The experimental data were analyzed using the Langmuir (Eq. 1), Freundlich (Eq. 2), Temkin (Eq. 3), and Sips (Eq. 4) isotherm models:

1 $${q}_{e}=\frac{{Q}_{m}{K}_{L}{C}_{e}}{1+\,{K}_{L}\,{C}_{e}}$$

2 $${q}_{e}={K}_{F}\,{C}_{e}^{1/n}\,$$

3 $${q}_{e}=B\,\mathrm{ln}\,A+B\,\mathrm{ln}\,{C}_{e};\,b=\frac{RT}{B}\,$$

4 $${q}_{e}=\frac{{Q}_{m}{K}_{L}{C}_{e}^{1/n}}{1+\,{K}_{L}\,{C}_{e}^{1/n}}$$

where *C*_*e*_ is the equilibrium concentration of chromium in the aqueous solution, *K*_*F*_ is the Freundlich constant related to the adsorption amount, *1/n* is the Freundlich constant related to the adsorption intensity, *Q*_*m*_ is the maximum sorption amount, and *K*_*L*_ is the Langmuir constant related to the affinity of the binding sites. In addition, *A* and *B* are the Temkin isotherm constant (L/g) and heat of sorption (J/mol), respectively. *R* is the sorption constant (J/mol/K) and *b* is the Temkin isotherm constant linked to the energy parameter, *B*. Equation 4 is equivalent to the Langmuir equation when *1/n* is equal to 1. Alternatively, as either *C*_*e*_ or *K*_*L*_ approach 0, this isotherm becomes equivalent to the Freundlich isotherm.

### Data availability

All data generated or analyzed during this study are included in this published article.

## Electronic supplementary material

Supplementary Information
