# Supplementary material for: Investigation of the mechanism of chromium removal in (3-aminopropyl)trimethoxysilane functionalized mesoporous silica
Source: Sci Rep. 2018 Aug 13;8:12078. doi: 10.1038/s41598-018-29679-x (PMC6089875; doi:10.1038/s41598-018-29679-x)
Supplement: Supplementary file 1 — Supplementary Information [file 41598_2018_29679_MOESM1_ESM.docx]

**Supplementary Material**

**Investigation of the mechanism of chromium removal in (3-aminopropyl)trimethoxysilane functionalized mesoporous silica**

JinHyeong Lee^1,†^, Jae-Hyun Kim^2,†^, Keunsu Choi^3,†^, Hee-Gon Kim^2,4^, Jeong-Ann Park^2^, So-Hye Cho^1,5^, Seok Won Hong^2,6^, Jung-Hyun Lee^7^, Jun Hee Lee^3^, Soonjae Lee^8,*^, Seung Yong Lee^1,5,*^, Jae-Woo Choi^2,6,*^

^1^ Materials Architecturing Research Center, Korea Institute of Science and Technology, Hwarang-ro 14-gil 5, Seongbuk-gu, Seoul 02792, Republic of Korea

^2^ Center for Water Resource Cycle Research, Korea Institute of Science and Technology, Hwarang-ro 14-gil 5, Seongbuk-gu, Seoul 02792, Republic of Korea

^3^ School of Energy and Chemical Engineering, Ulsan National Institute of Science and Technology, UNIST-gil, Ulsan 44919, Republic of Korea

^4^ Department of Chemical and Biological Engineering, Korea University, 145 Anam-ro, Seongbuk-gu, Seoul 02841, Republic of Korea

^5^ Division of Nano & Information Technology, KIST school, Korea University of Science and Technology, Hwarang-ro 14-gil 5, Seongbuk-gu, Seoul 02792, Republic of Korea

^6^ Division of Energy & Environment Technology, KIST school, Korea University of Science and Technology, Hwarang-ro 14-gil 5, Seongbuk-gu, Seoul 02792, Republic of Korea

^7^ Department of Chemical and Biological Engineering, Korea University, 145, Anam-ro, Seongbuk-gu, Seoul 02841, Republic of Korea

^8^ Department of Earth and Environmental Sciences, Korea University, 145, Anam-ro, Seongbuk-gu, Seoul 02841, Republic of Korea

* Corresponding. soonjam@korea.ac.kr (S. Lee), [patra@kist.re.kr](mailto:patra@kist.re.kr) (S.-Y. Lee), [plead36@kist.re.kr](mailto:plead36@kist.re.kr) (J.-W. Choi).

^†^ These authors contributed equally to this work

**Table S1.** Comparison of porous adsorbents for chromium removal.

| Adsorbent | pH | Adsorption time | Model used to calculated adsorption capacity | Adsorption capacity (mg/g) | ref |
| --- | --- | --- | --- | --- | --- |
| This study | 2.9 |  | Langmuir model | 84.9 |  |
| N-MCM-41 | 8-9 | 5 h | - | 52.9 | [1](#_ENREF_1) |
| N-SBA-1 | 8-9 | 5 h | - | 94.2 | [1](#_ENREF_1) |
| HAS-activated carbon(CK-22) | 3.2 | 5 days | Freundlich model | 73.9 | [2](#_ENREF_2) |
| N-doped porous carbon with magnetic particles | 3 | 10 min | Langmuir model | 16 | [3](#_ENREF_3) |
| Cellulose-montmorillonite composite | 3.8-5.5 | 40 min | Langmuir model | 22.2 | [4](#_ENREF_4) |
| Rice husk-based activated carbon (K4756) | 5.0 | 2 h | - | 34.7 | [5](#_ENREF_5) |
| γ-Fe_2_O_3_ | 2.5 | 120 min | - | 15.6 | [6](#_ENREF_6) |
| Chitosan coated on perlite | 4 | 180 min | Langmuir model | 153.8 | [7](#_ENREF_7) |





**Figure S1.** X-ray diffraction patterns for silica nanoparticles. The nanoparticles produce a single broad diffraction peak in the XRD pattern at 2*θ* value of 20–30° because of their amorphous nature^8, 9^.

**References**

1. Yoshitake, H. *et al.* Adsorption of chromate and arsenate by amino-functionalized MCM-41 and SBA-1. *Chem. Mat.* **14**(11), 4603-4610 (2002).

2. Hu, Z. et al. Chromium adsorption on high-performance activated carbons from aqueous solution. *Sep. Purif. Technol.* **31**(1), 13-18 (2003).

3. Li, Y. *et al.* N-doped porous carbon with magnetic particles formed in situ for enhanced Cr (VI) removal. *Water res.* **47**(12), 4188-4197 (2013).

4. Kumar, A.S.K. *et al.* Application of cellulose-clay composite biosorbent toward the effective adsorption and removal of chromium from industrial wastewater. *Ind. Eng. Chem. Res.* **51**(1), 58-69 (2011).

5. Guo, Y. *et al.* Adsorption of Cr (VI) on micro-and mesoporous rice husk-based active carbon. *Mater. Chem. Phys.* **78**(1), 132-137 (2003).

6. Wang, P. and Lo, I.M. Synthesis of mesoporous magnetic γ-Fe_2_O_3_ and its application to Cr (VI) removal from contaminated water. *Water res.* **43**(15), 3727-3734 (2009).

7. Hasan, S. *et al.* Adsorption of chromium (VI) on chitosan‐coated perlite. *Sep. sci. Technol.* **38**(15), 3775-3793 (2003).

8. Abdollahi, S. N. *et al.* Synthesis and physicochemical characterization of tunable silica-gold nanoshells via seed growth method. *Colloids Surf. A Physicochem. Eng. Asp.* **414**, 345–351 (2012).

9. Tadjarodi, A. *et al.* Experimental investigation of thermos-physical properties of platelet mesoporous SBA-15 silica particles dispersed in ethylene glycol and water mixture. *Ceram. Int.* **39**(7), 7649–7655 (2013).
